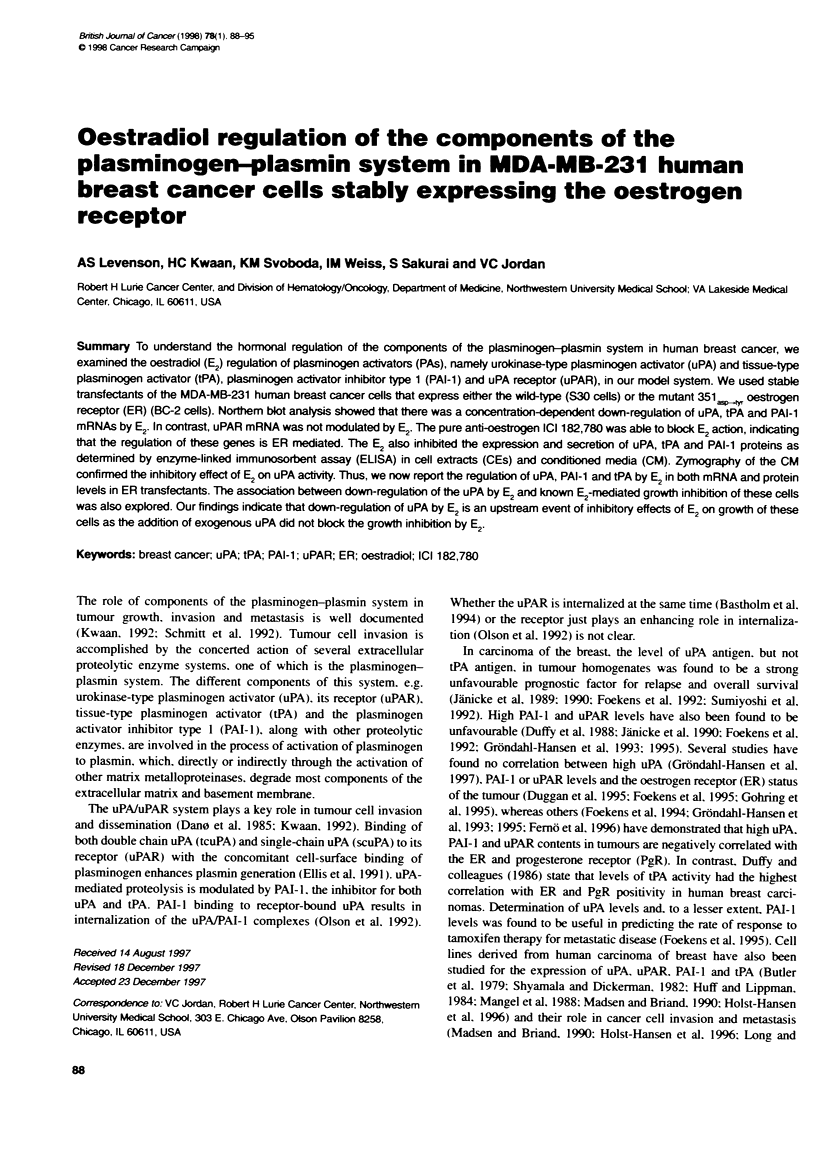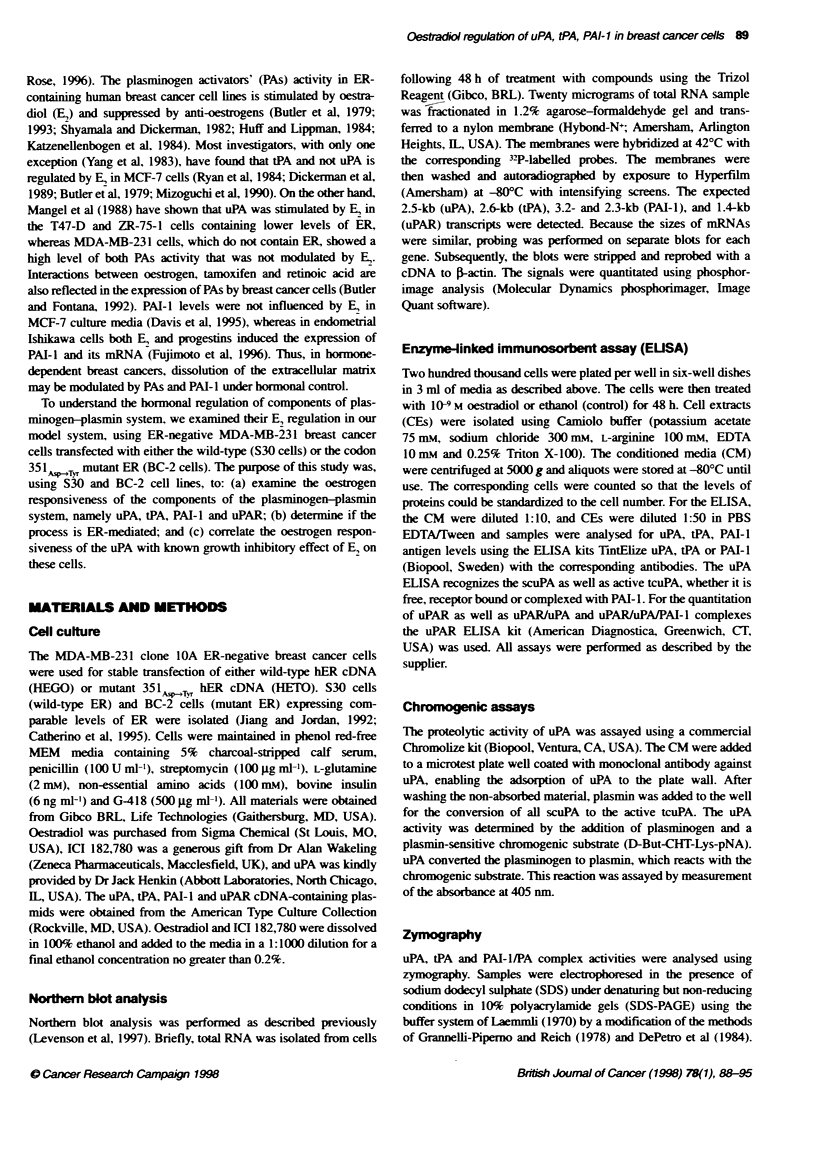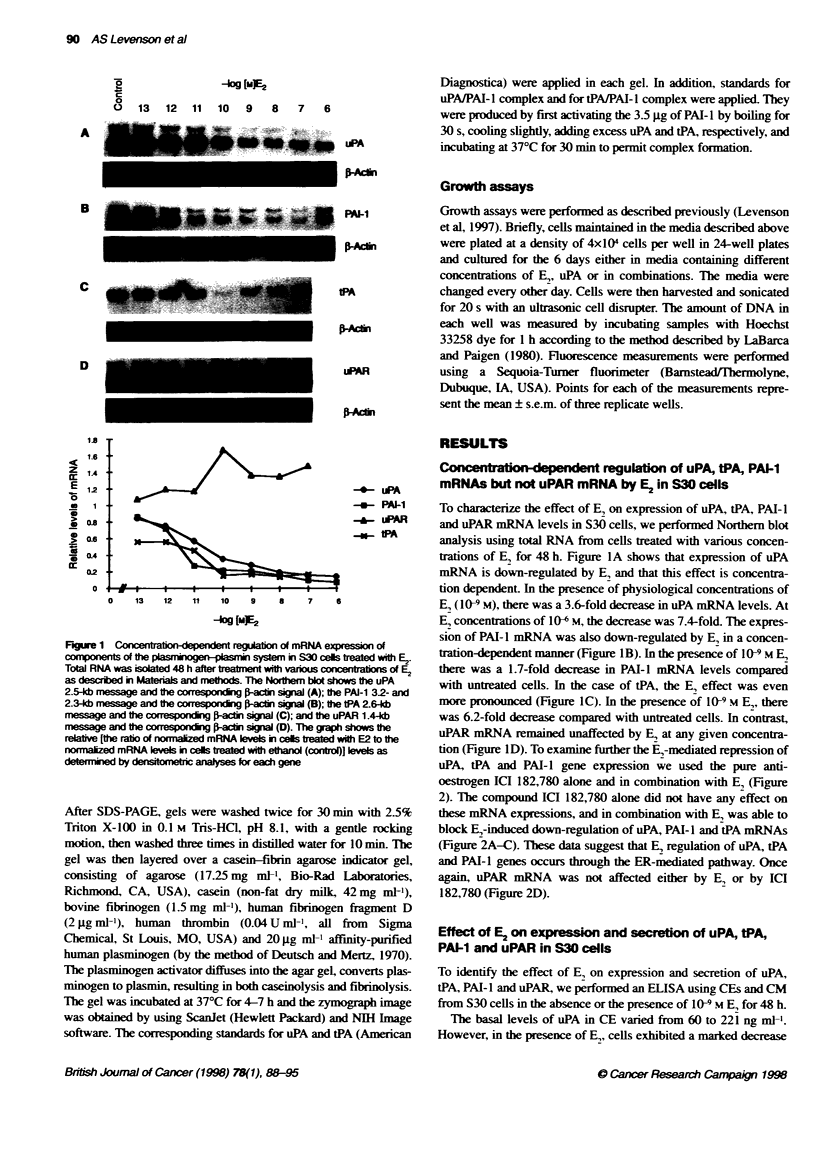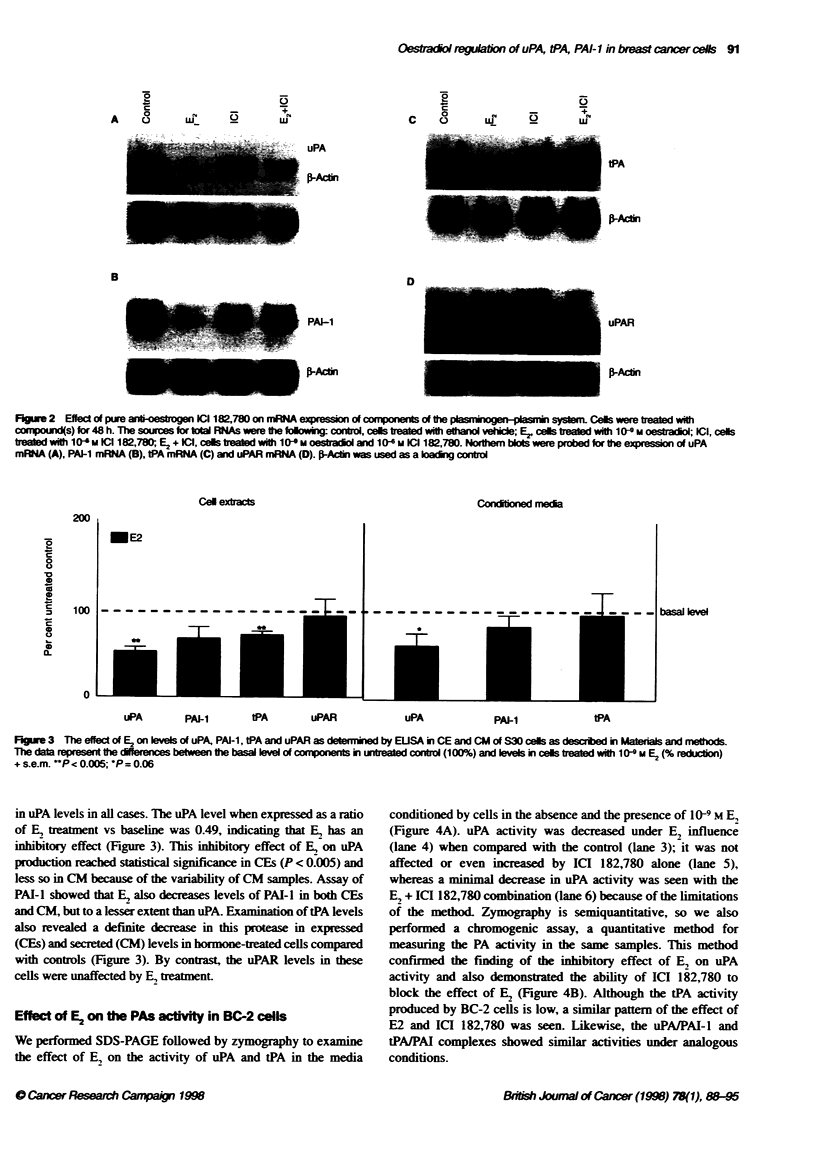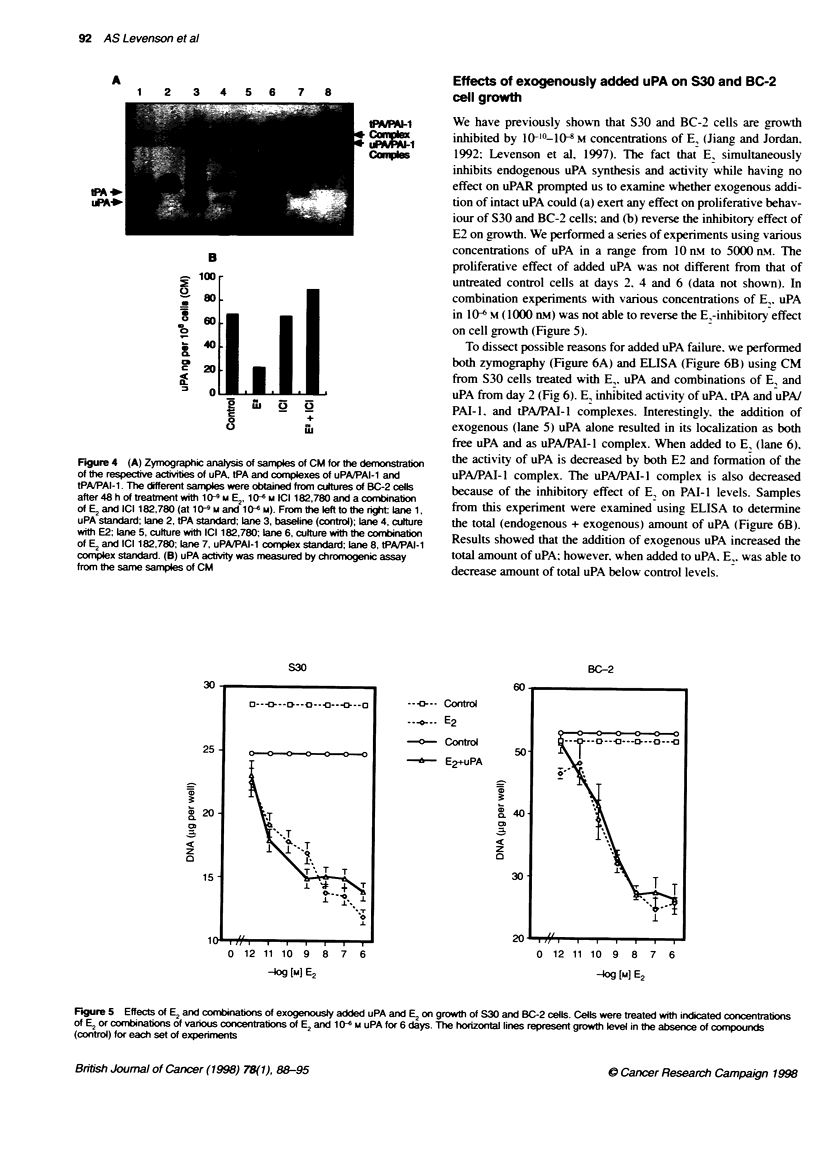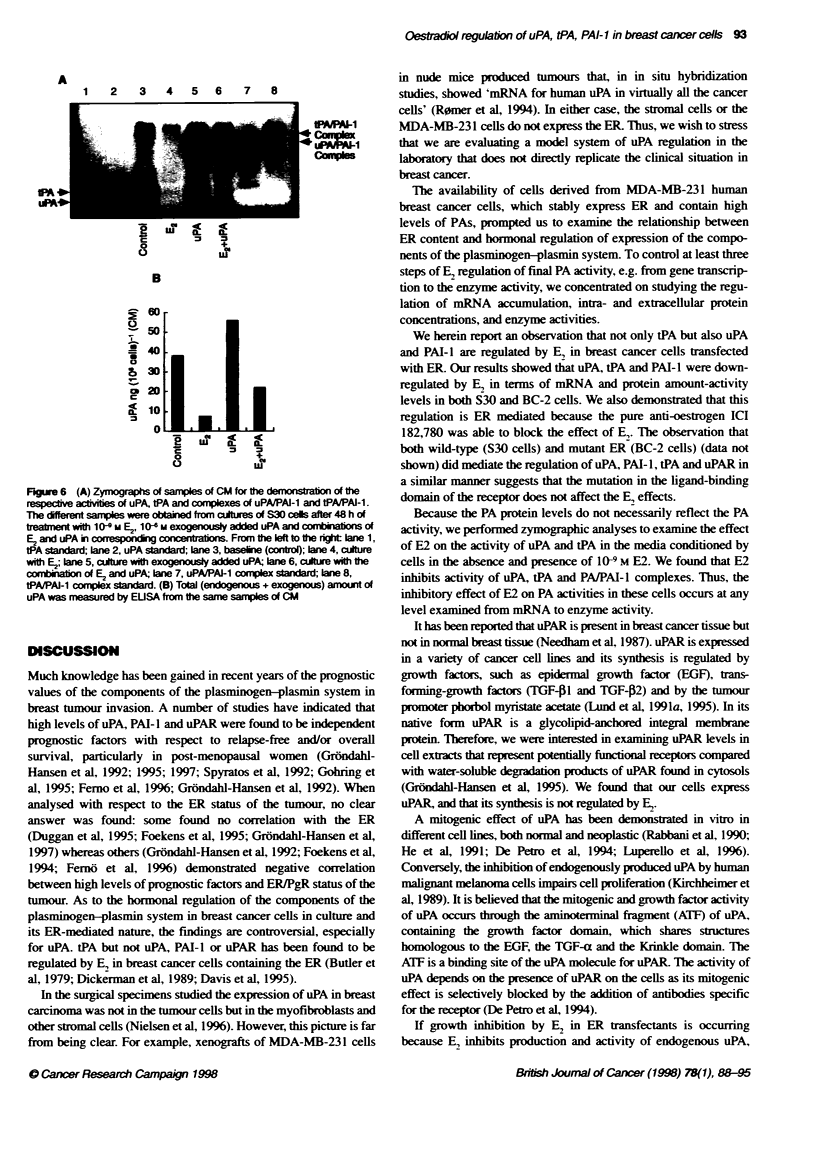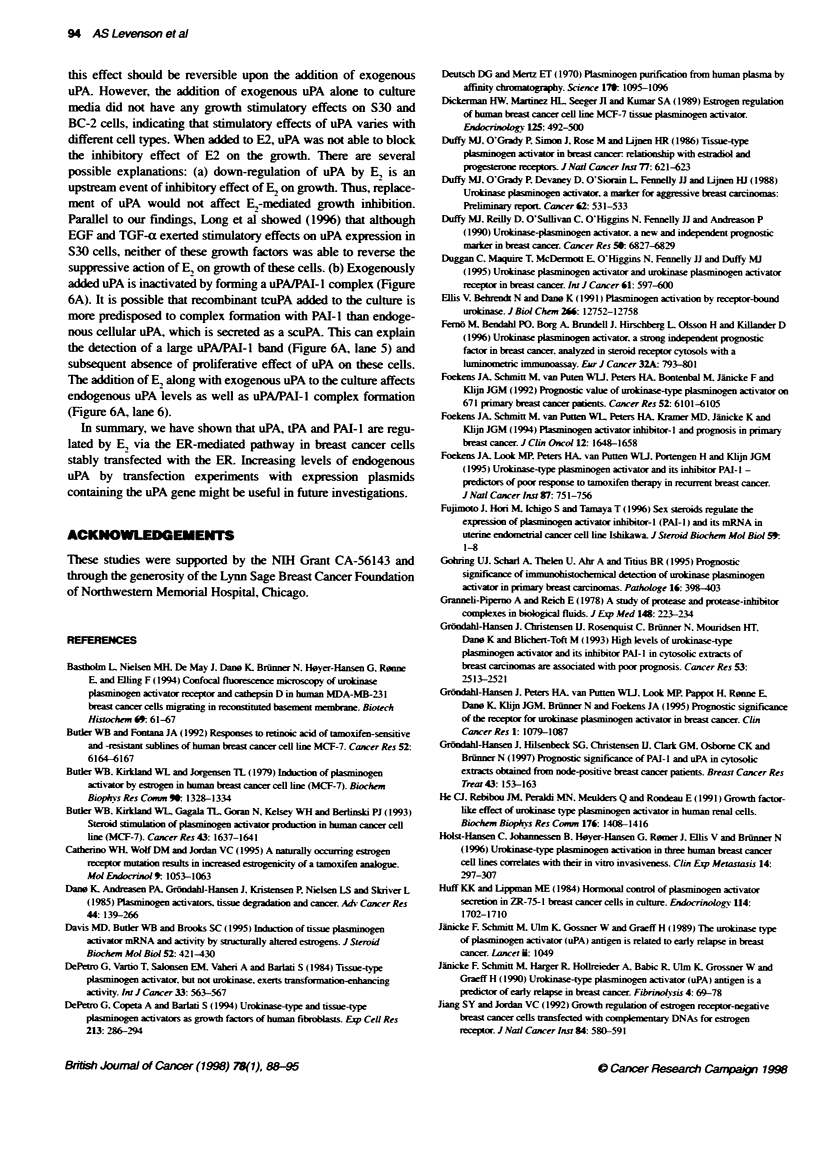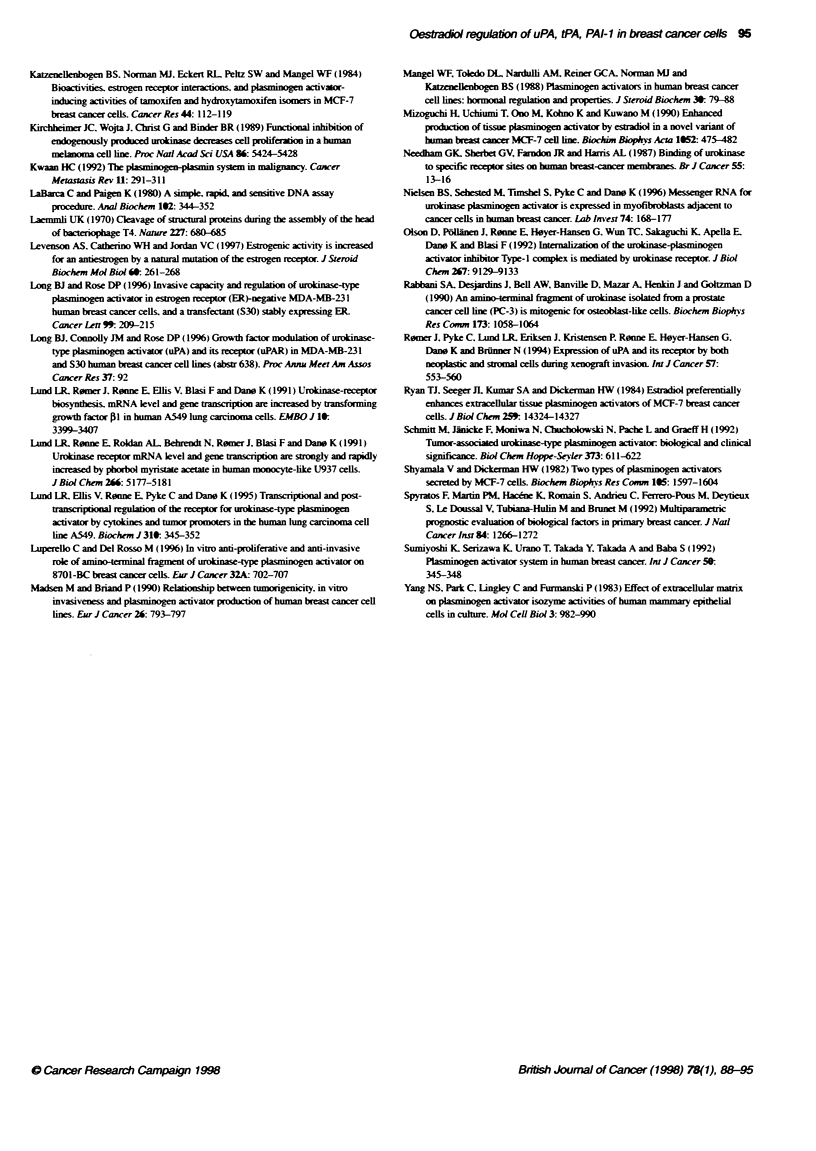# Oestradiol regulation of the components of the plasminogen-plasmin system in MDA-MB-231 human breast cancer cells stably expressing the oestrogen receptor.

**DOI:** 10.1038/bjc.1998.447

**Published:** 1998-07

**Authors:** A. S. Levenson, H. C. Kwaan, K. M. Svoboda, I. M. Weiss, S. Sakurai, V. C. Jordan

**Affiliations:** Robert H Lurie Cancer Center, Department of Medicine, Northwestern University Medical School, Chicago, IL 60611, USA.

## Abstract

**Images:**


British Journal of Cancer (1998) 78(1), 88–95
© 1998 Cancer Research Campaign

### Oestradiol regulation of the components of the plasminogen–plasmin system in MDA-MB-231 human breast cancer cells stably expressing the oestrogen receptor

**AS Levenson, HC Kwaan, KM Svoboda, IM Weiss, S Sakurai and VC Jordan**

Robert H Lurie Cancer Center, and Division of Hematology/Oncology, Department of Medicine, Northwestern University Medical School; VA Lakeside Medical Center, Chicago, IL 60611, USA

**Summary** To understand the hormonal regulation of the components of the plasminogen–plasmin system in human breast cancer, we examined the oestradiol ($E_2$) regulation of plasminogen activators (PAs), namely urokinase-type plasminogen activator (uPA) and tissue-type plasminogen activator (tPA), plasminogen activator inhibitor type 1 (PAI-1) and uPA receptor (uPAR), in our model system. We used stable transfectants of the MDA-MB-231 human breast cancer cells that express either the wild-type (S30 cells) or the mutant $351_{asp\rightarrow tyr}$ oestrogen receptor (ER) (BC-2 cells). Northern blot analysis showed that there was a concentration-dependent down-regulation of uPA, tPA and PAI-1 mRNAs by $E_2$. In contrast, uPAR mRNA was not modulated by $E_2$. The pure anti-oestrogen ICI 182,780 was able to block $E_2$ action, indicating that the regulation of these genes is ER mediated. The $E_2$ also inhibited the expression and secretion of uPA, tPA and PAI-1 proteins as determined by enzyme-linked immunosorbent assay (ELISA) in cell extracts (CEs) and conditioned media (CM). Zymography of the CM confirmed the inhibitory effect of $E_2$ on uPA activity. Thus, we now report the regulation of uPA, PAI-1 and tPA by $E_2$ in both mRNA and protein levels in ER transfectants. The association between down-regulation of the uPA by $E_2$ and known $E_2$-mediated growth inhibition of these cells was also explored. Our findings indicate that down-regulation of uPA by $E_2$ is an upstream event of inhibitory effects of $E_2$ on growth of these cells as the addition of exogenous uPA did not block the growth inhibition by $E_2$.

**Keywords:** breast cancer; uPA; tPA; PAI-1; uPAR; ER; oestradiol; ICI 182,780

The role of components of the plasminogen–plasmin system in tumour growth, invasion and metastasis is well documented (Kwaan, 1992; Schmitt et al, 1992). Tumour cell invasion is accomplished by the concerted action of several extracellular proteolytic enzyme systems, one of which is the plasminogen–plasmin system. The different components of this system, e.g. urokinase-type plasminogen activator (uPA), its receptor (uPAR), tissue-type plasminogen activator (tPA) and the plasminogen activator inhibitor type 1 (PAI-1), along with other proteolytic enzymes, are involved in the process of activation of plasminogen to plasmin, which, directly or indirectly through the activation of other matrix metalloproteinases, degrade most components of the extracellular matrix and basement membrane.

The uPA/uPAR system plays a key role in tumour cell invasion and dissemination (Danø et al, 1985; Kwaan, 1992). Binding of both double chain uPA (tcuPA) and single-chain uPA (scuPA) to its receptor (uPAR) with the concomitant cell-surface binding of plasminogen enhances plasmin generation (Ellis et al, 1991). uPA-mediated proteolysis is modulated by PAI-1, the inhibitor for both uPA and tPA. PAI-1 binding to receptor-bound uPA results in internalization of the uPA/PAI-1 complexes (Olson et al, 1992).

*Received 14 August 1997*
*Revised 18 December 1997*
*Accepted 23 December 1997*

*Correspondence to:* VC Jordan, Robert H Lurie Cancer Center, Northwestern University Medical School, 303 E. Chicago Ave, Olson Pavilion 8258, Chicago, IL 60611, USA

Whether the uPAR is internalized at the same time (Bastholm et al, 1994) or the receptor just plays an enhancing role in internalization (Olson et al, 1992) is not clear.

In carcinoma of the breast, the level of uPA antigen, but not tPA antigen, in tumour homogenates was found to be a strong unfavourable prognostic factor for relapse and overall survival (Jänicke et al, 1989; 1990; Foekens et al, 1992; Sumiyoshi et al, 1992). High PAI-1 and uPAR levels have also been found to be unfavourable (Duffy et al, 1988; Jänicke et al, 1990; Foekens et al, 1992; Gröndahl-Hansen et al, 1993; 1995). Several studies have found no correlation between high uPA (Gröndahl-Hansen et al, 1997), PAI-1 or uPAR levels and the oestrogen receptor (ER) status of the tumour (Duggan et al, 1995; Foekens et al, 1995; Gohring et al, 1995), whereas others (Foekens et al, 1994; Gröndahl-Hansen et al, 1993; 1995; Fernö et al, 1996) have demonstrated that high uPA, PAI-1 and uPAR contents in tumours are negatively correlated with the ER and progesterone receptor (PgR). In contrast, Duffy and colleagues (1986) state that levels of tPA activity had the highest correlation with ER and PgR positivity in human breast carcinomas. Determination of uPA levels and, to a lesser extent, PAI-1 levels was found to be useful in predicting the rate of response to tamoxifen therapy for metastatic disease (Foekens et al, 1995). Cell lines derived from human carcinoma of breast have also been studied for the expression of uPA, uPAR, PAI-1 and tPA (Butler et al, 1979; Shyamala and Dickerman, 1982; Huff and Lippman, 1984; Mangel et al, 1988; Madsen and Briand, 1990; Holst-Hansen et al, 1996) and their role in cancer cell invasion and metastasis (Madsen and Briand, 1990; Holst-Hansen et al, 1996; Long and

Rose, 1996). The plasminogen activators' (PAs) activity in ER-containing human breast cancer cell lines is stimulated by oestradiol ($E_2$) and suppressed by anti-oestrogens (Butler et al, 1979; 1993; Shyamala and Dickerman, 1982; Huff and Lippman, 1984; Katzenellenbogen et al, 1984). Most investigators, with only one exception (Yang et al, 1983), have found that tPA and not uPA is regulated by $E_2$ in MCF-7 cells (Ryan et al, 1984; Dickerman et al, 1989; Butler et al, 1979; Mizoguchi et al, 1990). On the other hand, Mangel et al (1988) have shown that uPA was stimulated by $E_2$ in the T47-D and ZR-75-1 cells containing lower levels of ER, whereas MDA-MB-231 cells, which do not contain ER, showed a high level of both PAs activity that was not modulated by $E_2$. Interactions between oestrogen, tamoxifen and retinoic acid are also reflected in the expression of PAs by breast cancer cells (Butler and Fontana, 1992). PAI-1 levels were not influenced by $E_2$ in MCF-7 culture media (Davis et al, 1995), whereas in endometrial Ishikawa cells both $E_2$ and progestins induced the expression of PAI-1 and its mRNA (Fujimoto et al, 1996). Thus, in hormone-dependent breast cancers, dissolution of the extracellular matrix may be modulated by PAs and PAI-1 under hormonal control.

To understand the hormonal regulation of components of plasminogen–plasmin system, we examined their $E_2$ regulation in our model system, using ER-negative MDA-MB-231 breast cancer cells transfected with either the wild-type (S30 cells) or the codon $351_{Asp\rightarrow Tyr}$ mutant ER (BC-2 cells). The purpose of this study was, using S30 and BC-2 cell lines, to: (a) examine the oestrogen responsiveness of the components of the plasminogen–plasmin system, namely uPA, tPA, PAI-1 and uPAR; (b) determine if the process is ER-mediated; and (c) correlate the oestrogen responsiveness of the uPA with known growth inhibitory effect of $E_2$ on these cells.

### MATERIALS AND METHODS

### Cell culture

The MDA-MB-231 clone 10A ER-negative breast cancer cells were used for stable transfection of either wild-type hER cDNA (HEGO) or mutant $351_{Asp\rightarrow Tyr}$ hER cDNA (HETO). S30 cells (wild-type ER) and BC-2 cells (mutant ER) expressing comparable levels of ER were isolated (Jiang and Jordan, 1992; Catherino et al, 1995). Cells were maintained in phenol red-free MEM media containing 5% charcoal-stripped calf serum, penicillin (100 U $ml^{-1}$), streptomycin (100 µg $ml^{-1}$), L-glutamine (2 mM), non-essential amino acids (100 mM), bovine insulin (6 ng $ml^{-1}$) and G-418 (500 µg $ml^{-1}$). All materials were obtained from Gibco BRL, Life Technologies (Gaithersburg, MD, USA). Oestradiol was purchased from Sigma Chemical (St Louis, MO, USA), ICI 182,780 was a generous gift from Dr Alan Wakeling (Zeneca Pharmaceuticals, Macclesfield, UK), and uPA was kindly provided by Dr Jack Henkin (Abbott Laboratories, North Chicago, IL, USA). The uPA, tPA, PAI-1 and uPAR cDNA-containing plasmids were obtained from the American Type Culture Collection (Rockville, MD, USA). Oestradiol and ICI 182,780 were dissolved in 100% ethanol and added to the media in a 1:1000 dilution for a final ethanol concentration no greater than 0.2%.

### Northern blot analysis

Northern blot analysis was performed as described previously (Levenson et al, 1997). Briefly, total RNA was isolated from cells following 48 h of treatment with compounds using the Trizol Reagent (Gibco, BRL). Twenty micrograms of total RNA sample was fractionated in 1.2% agarose–formaldehyde gel and transferred to a nylon membrane (Hybond-N+; Amersham, Arlington Heights, IL, USA). The membranes were hybridized at 42°C with the corresponding $^{32}P$-labelled probes. The membranes were then washed and autoradiographed by exposure to Hyperfilm (Amersham) at –80°C with intensifying screens. The expected 2.5-kb (uPA), 2.6-kb (tPA), 3.2- and 2.3-kb (PAI-1), and 1.4-kb (uPAR) transcripts were detected. Because the sizes of mRNAs were similar, probing was performed on separate blots for each gene. Subsequently, the blots were stripped and reprobed with a cDNA to β-actin. The signals were quantitated using phosphorimage analysis (Molecular Dynamics phosphorimager, Image Quant software).

### Enzyme-linked immunosorbent assay (ELISA)

Two hundred thousand cells were plated per well in six-well dishes in 3 ml of media as described above. The cells were then treated with $10^{-9}$ M oestradiol or ethanol (control) for 48 h. Cell extracts (CEs) were isolated using Camiolo buffer (potassium acetate 75 mM, sodium chloride 300 mM, L-arginine 100 mM, EDTA 10 mM and 0.25% Triton X-100). The conditioned media (CM) were centrifuged at 5000 *g* and aliquots were stored at –80°C until use. The corresponding cells were counted so that the levels of proteins could be standardized to the cell number. For the ELISA, the CM were diluted 1:10, and CEs were diluted 1:50 in PBS EDTA/Tween and samples were analysed for uPA, tPA, PAI-1 antigen levels using the ELISA kits TintElize uPA, tPA or PAI-1 (Biopool, Sweden) with the corresponding antibodies. The uPA ELISA recognizes the scuPA as well as active tcuPA, whether it is free, receptor bound or complexed with PAI-1. For the quantitation of uPAR as well as uPAR/uPA and uPAR/uPA/PAI-1 complexes the uPAR ELISA kit (American Diagnostica, Greenwich, CT, USA) was used. All assays were performed as described by the supplier.

### Chromogenic assays

The proteolytic activity of uPA was assayed using a commercial Chromolize kit (Biopool, Ventura, CA, USA). The CM were added to a microtest plate well coated with monoclonal antibody against uPA, enabling the adsorption of uPA to the plate wall. After washing the non-absorbed material, plasmin was added to the well for the conversion of all scuPA to the active tcuPA. The uPA activity was determined by the addition of plasminogen and a plasmin-sensitive chromogenic substrate (D-But-CHT-Lys-pNA). uPA converted the plasminogen to plasmin, which reacts with the chromogenic substrate. This reaction was assayed by measurement of the absorbance at 405 nm.

### Zymography uPA, tPA and PAI-1/PA complex activities were analysed using zymography. Samples were electrophoresed in the presence of sodium dodecyl sulphate (SDS) under denaturing but non-reducing conditions in 10% polyacrylamide gels (SDS-PAGE) using the buffer system of Laemmli (1970) by a modification of the methods of Grannelli-Piperno and Reich (1978) and DePetro et al (1984).

© Cancer Research Campaign 1998 

**Figure 1** Concentration-dependent regulation of mRNA expression of components of the plasminogen–plasmin system in S30 cells treated with $E_2$. Total RNA was isolated 48 h after treatment with various concentrations of $E_2$ as described in Materials and methods. The Northern blot shows the uPA 2.5-kb message and the corresponding β-actin signal (**A**); the PAI-1 3.2- and 2.3-kb message and the corresponding β-actin signal (**B**); the tPA 2.6-kb message and the corresponding β-actin signal (**C**); and the uPAR 1.4-kb message and the corresponding β-actin signal (**D**). The graph shows the relative [the ratio of normalized mRNA levels in cells treated with E2 to the normalized mRNA levels in cells treated with ethanol (control)] levels as determined by densitometric analyses for each gene

After SDS-PAGE, gels were washed twice for 30 min with 2.5% Triton X-100 in 0.1 M Tris-HCl, pH 8.1, with a gentle rocking motion, then washed three times in distilled water for 10 min. The gel was then layered over a casein–fibrin agarose indicator gel, consisting of agarose (17.25 mg $ml^{-1}$, Bio-Rad Laboratories, Richmond, CA, USA), casein (non-fat dry milk, 42 mg $ml^{-1}$), bovine fibrinogen (1.5 mg $ml^{-1}$), human fibrinogen fragment D (2 μg $ml^{-1}$), human thrombin (0.04 U $ml^{-1}$, all from Sigma Chemical, St Louis, MO, USA) and 20 μg $ml^{-1}$ affinity-purified human plasminogen (by the method of Deutsch and Mertz, 1970). The plasminogen activator diffuses into the agar gel, converts plasminogen to plasmin, resulting in both caseinolysis and fibrinolysis. The gel was incubated at 37°C for 4–7 h and the zymograph image was obtained by using ScanJet (Hewlett Packard) and NIH Image software. The corresponding standards for uPA and tPA (American Diagnostica) were applied in each gel. In addition, standards for uPA/PAI-1 complex and for tPA/PAI-1 complex were applied. They were produced by first activating the 3.5 μg of PAI-1 by boiling for 30 s, cooling slightly, adding excess uPA and tPA, respectively, and incubating at 37°C for 30 min to permit complex formation.

### Growth assays

Growth assays were performed as described previously (Levenson et al, 1997). Briefly, cells maintained in the media described above were plated at a density of $4{\times}10^4$ cells per well in 24-well plates and cultured for the 6 days either in media containing different concentrations of $E_2$, uPA or in combinations. The media were changed every other day. Cells were then harvested and sonicated for 20 s with an ultrasonic cell disrupter. The amount of DNA in each well was measured by incubating samples with Hoechst 33258 dye for 1 h according to the method described by LaBarca and Paigen (1980). Fluorescence measurements were performed using a Sequoia-Turner fluorimeter (Barnstead/Thermolyne, Dubuque, IA, USA). Points for each of the measurements represent the mean ± s.e.m. of three replicate wells.

### RESULTS

### Concentration-dependent regulation of uPA, tPA, PAI-1 mRNAs but not uPAR mRNA by $E_2$ in S30 cells

To characterize the effect of $E_2$ on expression of uPA, tPA, PAI-1 and uPAR mRNA levels in S30 cells, we performed Northern blot analysis using total RNA from cells treated with various concentrations of $E_2$ for 48 h. Figure 1A shows that expression of uPA mRNA is down-regulated by $E_2$ and that this effect is concentration dependent. In the presence of physiological concentrations of $E_2$ ($10^{-9}$ M), there was a 3.6-fold decrease in uPA mRNA levels. At $E_2$ concentrations of $10^{-6}$ M, the decrease was 7.4-fold. The expression of PAI-1 mRNA was also down-regulated by $E_2$ in a concentration-dependent manner (Figure 1B). In the presence of $10^{-9}$ M $E_2$ there was a 1.7-fold decrease in PAI-1 mRNA levels compared with untreated cells. In the case of tPA, the $E_2$ effect was even more pronounced (Figure 1C). In the presence of $10^{-9}$ M $E_2$, there was 6.2-fold decrease compared with untreated cells. In contrast, uPAR mRNA remained unaffected by $E_2$ at any given concentration (Figure 1D). To examine further the $E_2$-mediated repression of uPA, tPA and PAI-1 gene expression we used the pure anti-oestrogen ICI 182,780 alone and in combination with $E_2$ (Figure 2). The compound ICI 182,780 alone did not have any effect on these mRNA expressions, and in combination with $E_2$ was able to block $E_2$-induced down-regulation of uPA, PAI-1 and tPA mRNAs (Figure 2A–C). These data suggest that $E_2$ regulation of uPA, tPA and PAI-1 genes occurs through the ER-mediated pathway. Once again, uPAR mRNA was not affected either by $E_2$ or by ICI 182,780 (Figure 2D).

### Effect of $E_2$ on expression and secretion of uPA, tPA, PAI-1 and uPAR in S30 cells

To identify the effect of $E_2$ on expression and secretion of uPA, tPA, PAI-1 and uPAR, we performed an ELISA using CEs and CM from S30 cells in the absence or the presence of $10^{-9}$ M $E_2$ for 48 h.

The basal levels of uPA in CE varied from 60 to 221 ng $ml^{-1}$. However, in the presence of $E_2$, cells exhibited a marked decrease

© Cancer Research Campaign 1998

**Figure 3** The effect of $E_2$ on levels of uPA, PAI-1, tPA and uPAR as determined by ELISA in CE and CM of S30 cells as described in Materials and methods. The data represent the differences between the basal level of components in untreated control (100%) and levels in cells treated with $10^{-9}$ M $E_2$ (% reduction) + s.e.m. $^{**}P < 0.005$; $^{*}P = 0.06$

in uPA levels in all cases. The uPA level when expressed as a ratio of $E_2$ treatment vs baseline was 0.49, indicating that $E_2$ has an inhibitory effect (Figure 3). This inhibitory effect of $E_2$ on uPA production reached statistical significance in CEs ($P < 0.005$) and less so in CM because of the variability of CM samples. Assay of PAI-1 showed that $E_2$ also decreases levels of PAI-1 in both CEs and CM, but to a lesser extent than uPA. Examination of tPA levels also revealed a definite decrease in this protease in expressed (CEs) and secreted (CM) levels in hormone-treated cells compared with controls (Figure 3). By contrast, the uPAR levels in these cells were unaffected by $E_2$ treatment.

### Effect of $E_2$ on the PAs activity in BC-2 cells

We performed SDS-PAGE followed by zymography to examine the effect of $E_2$ on the activity of uPA and tPA in the media conditioned by cells in the absence and the presence of $10^{-9}$ M $E_2$ (Figure 4A). uPA activity was decreased under $E_2$ influence (lane 4) when compared with the control (lane 3); it was not affected or even increased by ICI 182,780 alone (lane 5), whereas a minimal decrease in uPA activity was seen with the $E_2$ + ICI 182,780 combination (lane 6) because of the limitations of the method. Zymography is semiquantitative, so we also performed a chromogenic assay, a quantitative method for measuring the PA activity in the same samples. This method confirmed the finding of the inhibitory effect of $E_2$ on uPA activity and also demonstrated the ability of ICI 182,780 to block the effect of $E_2$ (Figure 4B). Although the tPA activity produced by BC-2 cells is low, a similar pattern of the effect of E2 and ICI 182,780 was seen. Likewise, the uPA/PAI-1 and tPA/PAI complexes showed similar activities under analogous conditions.

© Cancer Research Campaign 1998 

**Figure 4** (**A**) Zymographic analysis of samples of CM for the demonstration of the respective activities of uPA, tPA and complexes of uPA/PAI-1 and tPA/PAI-1. The different samples were obtained from cultures of BC-2 cells after 48 h of treatment with $10^{-9}$ M $E_2$, $10^{-6}$ M ICI 182,780 and a combination of $E_2$ and ICI 182,780 (at $10^{-9}$ M and $10^{-6}$ M). From the left to the right: lane 1, uPA standard; lane 2, tPA standard; lane 3, baseline (control); lane 4, culture with E2; lane 5, culture with ICI 182,780; lane 6, culture with the combination of $E_2$ and ICI 182,780; lane 7, uPA/PAI-1 complex standard; lane 8, tPA/PAI-1 complex standard. (**B**) uPA activity was measured by chromogenic assay from the same samples of CM

### Effects of exogenously added uPA on S30 and BC-2 cell growth

We have previously shown that S30 and BC-2 cells are growth inhibited by $10^{-10}$–$10^{-8}$ M concentrations of $E_2$ (Jiang and Jordan, 1992; Levenson et al, 1997). The fact that $E_2$ simultaneously inhibits endogenous uPA synthesis and activity while having no effect on uPAR prompted us to examine whether exogenous addition of intact uPA could (a) exert any effect on proliferative behaviour of S30 and BC-2 cells; and (b) reverse the inhibitory effect of E2 on growth. We performed a series of experiments using various concentrations of uPA in a range from 10 nM to 5000 nM. The proliferative effect of added uPA was not different from that of untreated control cells at days 2, 4 and 6 (data not shown). In combination experiments with various concentrations of $E_2$, uPA in $10^{-6}$ M (1000 nM) was not able to reverse the $E_2$-inhibitory effect on cell growth (Figure 5).

To dissect possible reasons for added uPA failure, we performed both zymography (Figure 6A) and ELISA (Figure 6B) using CM from S30 cells treated with $E_2$, uPA and combinations of $E_2$ and uPA from day 2 (Fig 6). $E_2$ inhibited activity of uPA, tPA and uPA/PAI-1, and tPA/PAI-1 complexes. Interestingly, the addition of exogenous (lane 5) uPA alone resulted in its localization as both free uPA and as uPA/PAI-1 complex. When added to $E_2$ (lane 6), the activity of uPA is decreased by both E2 and formation of the uPA/PAI-1 complex. The uPA/PAI-1 complex is also decreased because of the inhibitory effect of $E_2$ on PAI-1 levels. Samples from this experiment were examined using ELISA to determine the total (endogenous + exogenous) amount of uPA (Figure 6B). Results showed that the addition of exogenous uPA increased the total amount of uPA; however, when added to uPA, $E_2$, was able to decrease amount of total uPA below control levels.

**Figure 5** Effects of $E_2$ and combinations of exogenously added uPA and $E_2$ on growth of S30 and BC-2 cells. Cells were treated with indicated concentrations of $E_2$ or combinations of various concentrations of $E_2$ and $10^{-6}$ M uPA for 6 days. The horizontal lines represent growth level in the absence of compounds (control) for each set of experiments

© Cancer Research Campaign 1998

**Figure 6** (**A**) Zymographs of samples of CM for the demonstration of the respective activities of uPA, tPA and complexes of uPA/PAI-1 and tPA/PAI-1. The different samples were obtained from cultures of S30 cells after 48 h of treatment with $10^{-9}$ M $E_2$, $10^{-6}$ M exogenously added uPA and combinations of $E_2$ and uPA in corresponding concentrations. From the left to the right: lane 1, tPA standard; lane 2, uPA standard; lane 3, baseline (control); lane 4, culture with $E_2$; lane 5, culture with exogenously added uPA; lane 6, culture with the combination of $E_2$ and uPA; lane 7, uPA/PAI-1 complex standard; lane 8, tPA/PAI-1 complex standard. (**B**) Total (endogenous + exogenous) amount of uPA was measured by ELISA from the same samples of CM

### DISCUSSION

Much knowledge has been gained in recent years of the prognostic values of the components of the plasminogen–plasmin system in breast tumour invasion. A number of studies have indicated that high levels of uPA, PAI-1 and uPAR were found to be independent prognostic factors with respect to relapse-free and/or overall survival, particularly in post-menopausal women (Gröndahl-Hansen et al, 1992; 1995; 1997; Spyratos et al, 1992; Gohring et al, 1995; Ferno et al, 1996; Gröndahl-Hansen et al, 1992). When analysed with respect to the ER status of the tumour, no clear answer was found: some found no correlation with the ER (Duggan et al, 1995; Foekens et al, 1995; Gröndahl-Hansen et al, 1997) whereas others (Gröndahl-Hansen et al, 1992; Foekens et al, 1994; Fernö et al, 1996) demonstrated negative correlation between high levels of prognostic factors and ER/PgR status of the tumour. As to the hormonal regulation of the components of the plasminogen–plasmin system in breast cancer cells in culture and its ER-mediated nature, the findings are controversial, especially for uPA. tPA but not uPA, PAI-1 or uPAR has been found to be regulated by $E_2$ in breast cancer cells containing the ER (Butler et al, 1979; Dickerman et al, 1989; Davis et al, 1995).

In the surgical specimens studied the expression of uPA in breast carcinoma was not in the tumour cells but in the myofibroblasts and other stromal cells (Nielsen et al, 1996). However, this picture is far from being clear. For example, xenografts of MDA-MB-231 cells in nude mice produced tumours that, in in situ hybridization studies, showed 'mRNA for human uPA in virtually all the cancer cells' (Rømer et al, 1994). In either case, the stromal cells or the MDA-MB-231 cells do not express the ER. Thus, we wish to stress that we are evaluating a model system of uPA regulation in the laboratory that does not directly replicate the clinical situation in breast cancer.

The availability of cells derived from MDA-MB-231 human breast cancer cells, which stably express ER and contain high levels of PAs, prompted us to examine the relationship between ER content and hormonal regulation of expression of the components of the plasminogen–plasmin system. To control at least three steps of $E_2$ regulation of final PA activity, e.g. from gene transcription to the enzyme activity, we concentrated on studying the regulation of mRNA accumulation, intra- and extracellular protein concentrations, and enzyme activities.

We herein report an observation that not only tPA but also uPA and PAI-1 are regulated by $E_2$ in breast cancer cells transfected with ER. Our results showed that uPA, tPA and PAI-1 were down-regulated by $E_2$ in terms of mRNA and protein amount-activity levels in both S30 and BC-2 cells. We also demonstrated that this regulation is ER mediated because the pure anti-oestrogen ICI 182,780 was able to block the effect of $E_2$. The observation that both wild-type (S30 cells) and mutant ER (BC-2 cells) (data not shown) did mediate the regulation of uPA, PAI-1, tPA and uPAR in a similar manner suggests that the mutation in the ligand-binding domain of the receptor does not affect the $E_2$ effects.

Because the PA protein levels do not necessarily reflect the PA activity, we performed zymographic analyses to examine the effect of E2 on the activity of uPA and tPA in the media conditioned by cells in the absence and presence of $10^{-9}$ M E2. We found that E2 inhibits activity of uPA, tPA and PA/PAI-1 complexes. Thus, the inhibitory effect of E2 on PA activities in these cells occurs at any level examined from mRNA to enzyme activity.

It has been reported that uPAR is present in breast cancer tissue but not in normal breast tissue (Needham et al, 1987). uPAR is expressed in a variety of cancer cell lines and its synthesis is regulated by growth factors, such as epidermal growth factor (EGF), transforming-growth factors (TGF-β1 and TGF-β2) and by the tumour promoter phorbol myristate acetate (Lund et al, 1991*a*, 1995). In its native form uPAR is a glycolipid-anchored integral membrane protein. Therefore, we were interested in examining uPAR levels in cell extracts that represent potentially functional receptors compared with water-soluble degradation products of uPAR found in cytosols (Gröndahl-Hansen et al, 1995). We found that our cells express uPAR, and that its synthesis is not regulated by $E_2$.

A mitogenic effect of uPA has been demonstrated in vitro in different cell lines, both normal and neoplastic (Rabbani et al, 1990; He et al, 1991; De Petro et al, 1994; Luperello et al, 1996). Conversely, the inhibition of endogenously produced uPA by human malignant melanoma cells impairs cell proliferation (Kirchheimer et al, 1989). It is believed that the mitogenic and growth factor activity of uPA occurs through the aminoterminal fragment (ATF) of uPA, containing the growth factor domain, which shares structures homologous to the EGF, the TGF-α and the Krinkle domain. The ATF is a binding site of the uPA molecule for uPAR. The activity of uPA depends on the presence of uPAR on the cells as its mitogenic effect is selectively blocked by the addition of antibodies specific for the receptor (De Petro et al, 1994).

If growth inhibition by $E_2$ in ER transfectants is occurring because $E_2$ inhibits production and activity of endogenous uPA,

© *Cancer Research Campaign 1998* 

this effect should be reversible upon the addition of exogenous uPA. However, the addition of exogenous uPA alone to culture media did not have any growth stimulatory effects on S30 and BC-2 cells, indicating that stimulatory effects of uPA varies with different cell types. When added to E2, uPA was not able to block the inhibitory effect of E2 on the growth. There are several possible explanations: (a) down-regulation of uPA by $E_2$ is an upstream event of inhibitory effect of $E_2$ on growth. Thus, replacement of uPA would not affect $E_2$-mediated growth inhibition. Parallel to our findings, Long et al showed (1996) that although EGF and TGF-α exerted stimulatory effects on uPA expression in S30 cells, neither of these growth factors was able to reverse the suppressive action of $E_2$ on growth of these cells. (b) Exogenously added uPA is inactivated by forming a uPA/PAI-1 complex (Figure 6A). It is possible that recombinant tcuPA added to the culture is more predisposed to complex formation with PAI-1 than endogenous cellular uPA, which is secreted as a scuPA. This can explain the detection of a large uPA/PAI-1 band (Figure 6A, lane 5) and subsequent absence of proliferative effect of uPA on these cells. The addition of $E_2$ along with exogenous uPA to the culture affects endogenous uPA levels as well as uPA/PAI-1 complex formation (Figure 6A, lane 6).

In summary, we have shown that uPA, tPA and PAI-1 are regulated by $E_2$ via the ER-mediated pathway in breast cancer cells stably transfected with the ER. Increasing levels of endogenous uPA by transfection experiments with expression plasmids containing the uPA gene might be useful in future investigations.

### ACKNOWLEDGEMENTS

These studies were supported by the NIH Grant CA-56143 and through the generosity of the Lynn Sage Breast Cancer Foundation of Northwestern Memorial Hospital, Chicago.

### REFERENCES

Bastholm L, Nielsen MH, De May J, Danø K, Brünner N, Høyer-Hansen G, Rønne E, and Elling F (1994) Confocal fluorescence microscopy of urokinase plasminogen activator receptor and cathepsin D in human MDA-MB-231 breast cancer cells migrating in reconstituted basement membrane. *Biotech Histochem* **69**: 61–67

Butler WB and Fontana JA (1992) Responses to retinoic acid of tamoxifen-sensitive and -resistant sublines of human breast cancer cell line MCF-7. *Cancer Res* **52**: 6164–6167

Butler WB, Kirkland WL and Jorgensen TL (1979) Induction of plasminogen activator by estrogen in human breast cancer cell line (MCF-7). *Biochem Biophys Res Comm* **90**: 1328–1334

Butler WB, Kirkland WL, Gagala TL, Goran N, Kelsey WH and Berlinski PJ (1993) Steroid stimulation of plasminogen activator production in human cancer cell line (MCF-7). *Cancer Res* **43**: 1637–1641

Catherino WH, Wolf DM and Jordan VC (1995) A naturally occurring estrogen receptor mutation results in increased estrogenicity of a tamoxifen analogue. *Mol Endocrinol* **9**: 1053–1063

Danø K, Andreasen PA, Gröndahl-Hansen J, Kristensen P, Nielsen LS and Skriver L (1985) Plasminogen activators, tissue degradation and cancer. *Adv Cancer Res* **44**: 139–266

Davis MD, Butler WB and Brooks SC (1995) Induction of tissue plasminogen activator mRNA and activity by structurally altered estrogens. *J Steroid Biochem Mol Biol* **52**: 421–430

DePetro G, Vartio T, Salonsen EM, Vaheri A and Barlati S (1984) Tissue-type plasminogen activator, but not urokinase, exerts transformation-enhancing activity. *Int J Cancer* **33**: 563–567

DePetro G, Copeta A and Barlati S (1994) Urokinase-type and tissue-type plasminogen activators as growth factors of human fibroblasts. *Exp Cell Res* **213**: 286–294

Deutsch DG and Mertz ET (1970) Plasminogen purification from human plasma by affinity chromatography. *Science* **170**: 1095–1096

Dickerman HW, Martinez HL, Seeger JI and Kumar SA (1989) Estrogen regulation of human breast cancer cell line MCF-7 tissue plasminogen activator. *Endocrinology* **125**: 492–500

Duffy MJ, O'Grady P, Simon J, Rose M and Lijnen HR (1986) Tissue-type plasminogen activator in breast cancer: relationship with estradiol and progesterone receptors. *J Natl Cancer Inst* **77**: 621–623

Duffy MJ, O'Grady P, Devaney D, O'Siorain L, Fennelly JJ and Lijnen HJ (1988) Urokinase plasminogen activator, a marker for aggressive breast carcinomas: Preliminary report. *Cancer* **62**: 531–533

Duffy MJ, Reilly D, O'Sullivan C, O'Higgins N, Fennelly JJ and Andreason P (1990) Urokinase-plasminogen activator, a new and independent prognostic marker in breast cancer. *Cancer Res* **50**: 6827–6829

Duggan C, Maquire T, McDermott E, O'Higgins N, Fennelly JJ and Duffy MJ (1995) Urokinase plasminogen activator and urokinase plasminogen activator receptor in breast cancer. *Int J Cancer* **61**: 597–600

Ellis V, Behrendt N and Danø K (1991) Plasminogen activation by receptor-bound urokinase. *J Biol Chem* **266**: 12752–12758

Fernö M, Bendahl PO, Borg A, Brundell J, Hirschberg L, Olsson H and Killander D (1996) Urokinase plasminogen activator, a strong independent prognostic factor in breast cancer, analyzed in steroid receptor cytosols with a luminometric immunoassay. *Eur J Cancer* **32A**: 793–801

Foekens JA, Schmitt M, van Puten WLJ, Peters HA, Bontenbal M, Jänicke F and Klijn JGM (1992) Prognostic value of urokinase-type plasminogen activator on 671 primary breast cancer patients. *Cancer Res* **52**: 6101–6105

Foekens JA, Schmitt M, van Putten WL, Peters HA, Kramer MD, Jänicke K and Klijn JGM (1994) Plasminogen activator inhibitor-1 and prognosis in primary breast cancer. *J Clin Oncol* **12**: 1648–1658

Foekens JA, Look MP, Peters HA, van Putten WLJ, Portengen H and Klijn JGM (1995) Urokinase-type plasminogen activator and its inhibitor PAI-1 – predictors of poor response to tamoxifen therapy in recurrent breast cancer. *J Natl Cancer Inst* **87**: 751–756

Fujimoto J, Hori M, Ichigo S and Tamaya T (1996) Sex steroids regulate the expression of plasminogen activator inhibitor-1 (PAI-1) and its mRNA in uterine endometrial cancer cell line Ishikawa. *J Steroid Biochem Mol Biol* **59**: 1–8

Gohring UJ, Scharl A, Thelen U, Ahr A and Titius BR (1995) Prognostic significance of immunohistochemical detection of urokinase plasminogen activator in primary breast carcinomas. *Pathologe* **16**: 398–403

Granneli-Piperno A and Reich E (1978) A study of protease and protease-inhibitor complexes in biological fluids. *J Exp Med* **148**: 223–234

Gröndahl-Hansen J, Christensen IJ, Rosenquist C, Brünner N, Mouridsen HT, Danø K and Blichert-Toft M (1993) High levels of urokinase-type plasminogen activator and its inhibitor PAI-1 in cytosolic extracts of breast carcinomas are associated with poor prognosis. *Cancer Res* **53**: 2513–2521

Gröndahl-Hansen J, Peters HA, van Putten WLJ, Look MP, Pappot H, Rønne E, Danø K, Klijn JGM, Brünner N and Foekens JA (1995) Prognostic significance of the receptor for urokinase plasminogen activator in breast cancer. *Clin Cancer Res* **1**: 1079–1087

Gröndahl-Hansen J, Hilsenbeck SG, Christensen IJ, Clark GM, Osborne CK and Brünner N (1997) Prognostic significance of PAI-1 and uPA in cytosolic extracts obtained from node-positive breast cancer patients. *Breast Cancer Res Treat* **43**: 153–163

He CJ, Rebibou JM, Peraldi MN, Meulders Q and Rondeau E (1991) Growth factor-like effect of urokinase type plasminogen activator in human renal cells. *Biochem Biophys Res Comm* **176**: 1408–1416

Holst-Hansen C, Johannessen B, Høyer-Hansen G, Rømer J, Ellis V and Brünner N (1996) Urokinase-type plasminogen activation in three human breast cancer cell lines correlates with their in vitro invasiveness. *Clin Exp Metastasis* **14**: 297–307

Huff KK and Lippman ME (1984) Hormonal control of plasminogen activator secretion in ZR-75-1 breast cancer cells in culture. *Endocrinology* **114**: 1702–1710

Jänicke F, Schmitt M, Ulm K, Gossner W and Graeff H (1989) The urokinase type of plasminogen activator (uPA) antigen is related to early relapse in breast cancer. *Lancet* **ii**: 1049

Jänicke F, Schmitt M, Harger R, Hollreieder A, Babic R, Ulm K, Grossner W and Graeff H (1990) Urokinase-type plasminogen activator (uPA) antigen is a predictor of early relapse in breast cancer. *Fibrinolysis* **4**: 69–78

Jiang SY and Jordan VC (1992) Growth regulation of estrogen receptor-negative breast cancer cells transfected with complementary DNAs for estrogen receptor. *J Natl Cancer Inst* **84**: 580–591

© Cancer Research Campaign 1998

Katzenellenbogen BS, Norman MJ, Eckert RL, Peltz SW and Mangel WF (1984) Bioactivities, estrogen receptor interactions, and plasminogen activator-inducing activities of tamoxifen and hydroxytamoxifen isomers in MCF-7 breast cancer cells. *Cancer Res* **44**: 112–119

Kirchheimer JC, Wojta J, Christ G and Binder BR (1989) Functional inhibition of endogenously produced urokinase decreases cell proliferation in a human melanoma cell line. *Proc Natl Acad Sci USA* **86**: 5424–5428

Kwaan HC (1992) The plasminogen-plasmin system in malignancy. *Cancer Metastasis Rev* **11**: 291–311

LaBarca C and Paigen K (1980) A simple, rapid, and sensitive DNA assay procedure. *Anal Biochem* **102**: 344–352

Laemmli UK (1970) Cleavage of structural proteins during the assembly of the head of bacteriophage T4. *Nature* **227**: 680–685

Levenson AS, Catherino WH and Jordan VC (1997) Estrogenic activity is increased for an antiestrogen by a natural mutation of the estrogen receptor. *J Steroid Biochem Mol Biol* **60**: 261–268

Long BJ and Rose DP (1996) Invasive capacity and regulation of urokinase-type plasminogen activator in estrogen receptor (ER)-negative MDA-MB-231 human breast cancer cells, and a transfectant (S30) stably expressing ER. *Cancer Lett* **99**: 209–215

Long BJ, Connolly JM and Rose DP (1996) Growth factor modulation of urokinase-type plasminogen activator (uPA) and its receptor (uPAR) in MDA-MB-231 and S30 human breast cancer cell lines (abstr 638). *Proc Annu Meet Am Assos Cancer Res* **37**: 92

Lund LR, Rømer J, Rønne E, Ellis V, Blasi F and Danø K (1991) Urokinase-receptor biosynthesis, mRNA level and gene transcription are increased by transforming growth factor β1 in human A549 lung carcinoma cells. *EMBO J* **10**: 3399–3407

Lund LR, Rønne E, Roldan AL, Behrendt N, Rømer J, Blasi F and Danø K (1991) Urokinase receptor mRNA level and gene transcription are strongly and rapidly increased by phorbol myristate acetate in human monocyte-like U937 cells. *J Biol Chem* **266**: 5177–5181

Lund LR, Ellis V, Rønne E, Pyke C and Danø K (1995) Transcriptional and post-transcriptional regulation of the receptor for urokinase-type plasminogen activator by cytokines and tumor promoters in the human lung carcinoma cell line A549. *Biochem J* **310**: 345–352

Luperello C and Del Rosso M (1996) In vitro anti-proliferative and anti-invasive role of amino-terminal fragment of urokinase-type plasminogen activator on 8701-BC breast cancer cells. *Eur J Cancer* **32A**: 702–707

Madsen M and Briand P (1990) Relationship between tumorigenicity, in vitro invasiveness and plasminogen activator production of human breast cancer cell lines. *Eur J Cancer* **26**: 793–797

Mangel WF, Toledo DL, Nardulli AM, Reiner GCA, Norman MJ and Katzenellenbogen BS (1988) Plasminogen activators in human breast cancer cell lines: hormonal regulation and properties. *J Steroid Biochem* **30**: 79–88

Mizoguchi H, Uchiumi T, Ono M, Kohno K and Kuwano M (1990) Enhanced production of tissue plasminogen activator by estradiol in a novel variant of human breast cancer MCF-7 cell line. *Biochim Biophys Acta* **1052**: 475–482

Needham GK, Sherbet GV, Farndon JR and Harris AL (1987) Binding of urokinase to specific receptor sites on human breast-cancer membranes. *Br J Cancer* **55**: 13–16

Nielsen BS, Sehested M, Timshel S, Pyke C and Danø K (1996) Messenger RNA for urokinase plasminogen activator is expressed in myofibroblasts adjacent to cancer cells in human breast cancer. *Lab Invest* **74**: 168–177

Olson D, Pöllänen J, Rønne E, Høyer-Hansen G, Wun TC, Sakaguchi K, Apella E, Danø K and Blasi F (1992) Internalization of the urokinase-plasminogen activator inhibitor Type-1 complex is mediated by urokinase receptor. *J Biol Chem* **267**: 9129–9133

Rabbani SA, Desjardins J, Bell AW, Banville D, Mazar A, Henkin J and Goltzman D (1990) An amino-terminal fragment of urokinase isolated from a prostate cancer cell line (PC-3) is mitogenic for osteoblast-like cells. *Biochem Biophys Res Comm* **173**: 1058–1064

Rømer J, Pyke C, Lund LR, Eriksen J, Kristensen P, Rønne E, Høyer-Hansen G, Danø K and Brünner N (1994) Expression of uPA and its receptor by both neoplastic and stromal cells during xenograft invasion. *Int J Cancer* **57**: 553–560

Ryan TJ, Seeger JI, Kumar SA and Dickerman HW (1984) Estradiol preferentially enhances extracellular tissue plasminogen activators of MCF-7 breast cancer cells. *J Biol Chem* **259**: 14324–14327

Schmitt M, Jänicke F, Moniwa N, Chucholowski N, Pache L and Graeff H (1992) Tumor-associated urokinase-type plasminogen activator: biological and clinical significance. *Biol Chem Hoppe-Seyler* **373**: 611–622

Shyamala V and Dickerman HW (1982) Two types of plasminogen activators secreted by MCF-7 cells. *Biochem Biophys Res Comm* **105**: 1597–1604

Spyratos F, Martin PM, Hacéne K, Romain S, Andrieu C, Ferrero-Pous M, Deytieux S, Le Doussal V, Tubiana-Hulin M and Brunet M (1992) Multiparametric prognostic evaluation of biological factors in primary breast cancer. *J Natl Cancer Inst* **84**: 1266–1272

Sumiyoshi K, Serizawa K, Urano T, Takada Y, Takada A and Baba S (1992) Plasminogen activator system in human breast cancer. *Int J Cancer* **50**: 345–348

Yang NS, Park C, Lingley C and Furmanski P (1983) Effect of extracellular matrix on plasminogen activator isozyme activities of human mammary epithelial cells in culture. *Mol Cell Biol* **3**: 982–990

© Cancer Research Campaign 1998